# Background Frequency Patterns in Standard Electroencephalography as an Early Prognostic Tool in Out-of-Hospital Cardiac Arrest Survivors Treated with Targeted Temperature Management

**DOI:** 10.3390/jcm9041113

**Published:** 2020-04-13

**Authors:** Youn-Jung Kim, Min-Jee Kim, Yong Seo Koo, Won Young Kim

**Affiliations:** 1 Department of Emergency Medicine, Asan Medical Center, Ulsan University College of Medicine, Seoul 05505, Korea; yjkim.em@gmail.com; 2 Department of Pediatrics, Asan Medical Center Children’s Hospital, Ulsan University College of Medicine, Seoul 05505, Korea; pradoxwh@naver.com; 3 Department of Neurology, Asan Medical Center, Ulsan University College of Medicine, Seoul 05505, Korea; yo904@amc.seoul.kr

**Keywords:** out-of-hospital cardiac arrest, electroencephalography, hypoxic brain damage, prognosis

## Abstract

We investigated the prognostic value of standard electroencephalography, a 30-min recording using 21 electrodes on the scalp, during the early post-cardiac arrest period, and evaluated the performance of electroencephalography findings combined with other clinical features for predicting favourable outcomes in comatose out-of-hospital cardiac arrest (OHCA) survivors treated with targeted temperature management (TTM). This observational registry-based study was conducted at a tertiary care hospital in Korea using the data of all consecutive adult non-traumatic comatose OHCA survivors who underwent standard electroencephalography during TTM between 2010 and 2018. The primary outcome was a 6-month favourable neurological outcome (Cerebral Performance Category score of 1 or 2). Among 170 comatose OHCA survivors with median electroencephalography time of 22 h, a 6-month favourable neurologic outcome was observed in 34.1% (58/170). After adjusting other clinical characteristics, an electroencephalography background with dominant alpha and theta waves had the highest odds ratio of 13.03 (95% confidence interval, 4.69–36.22) in multivariable logistic analysis. A combination of other clinical features (age < 65 years, initial shockable rhythm, resuscitation duration < 20 min) with an electroencephalography background with dominant alpha and theta waves increased predictive performance for favourable neurologic outcomes with a high specificity of up to 100%. A background with dominant alpha and theta waves in standard electroencephalography during TTM could be a simple and early favourable prognostic finding in comatose OHCA survivors.

## 1. Introduction

Hypoxic–ischaemic brain injury is common after resuscitation from cardiac arrest (CA). Most out-of-hospital cardiac arrest (OHCA) survivors are initially unconscious at hospital admission [1,2], and they undergo intensive post-resuscitation care, including targeted temperature management (TTM) [3,4]. Despite substantial allocations of medical resources, the overall outcomes of OHCA remain dismal, with only 8.3% survivors having a favourable neurological outcome and being eligible for discharge [5]; furthermore, withdrawal of life-sustaining therapy (WSLT), owing to a perceived unfavourable neurological prognosis is a leading cause of death [6,7,8]. A multimodal approach and delayed timing (after >72 h) of prognostication has been recommended to minimize the possibility of inaccurate WSLT for patients who demonstrate a change in neurological recovery [3,4]. However, one of the most pressing issues for relatives and healthcare workers is to rapidly obtain reliable information regarding the probability of achieving favourable neurological outcomes. Although numerous studies have focused on discovering factors for unfavourable neurologic outcomes [3,4], it is essential to develop strategies for predicting favourable neurologic outcomes among OHCA survivors in order to appropriately tailor medical therapies for each patient. 

Electroencephalography (EEG) in post-CA patients has been used for dual purposes—the detection of seizure activity in the early stages of TTM and prognostication of neurological outcomes. Persistent absence of EEG reactivity, intractable status epilepticus and persistent burst-suppression patterns at ≥72 h after CA are the suggested prognostic tools that indicate unfavourable neurologic outcomes in comatose CA patients in the international guidelines [3,4]. However, the use of different classification systems and inter-rater variability, including reproducibility and reliability, serve as the limitations of EEG as a prognostic tool [3,4,9]. To enhance the prognostic value of EEG, the American Clinical Neurophysiology Society (ACNS) recently proposed a standardized EEG terminology that can be suitably used for critically ill patients after CA [10]. Recent studies have reported the feasibility of using EEG as an early prognostic tool <72 h after CA for OHCA survivors [6,11,12,13]. Continuous EEG monitoring provides more real-time information than standard intermittent EEG; however, its requirement for an extensive amount of medical resources and timely unavailability are major limitations in clinical practice compared with standard intermittent EEG [9,12]. 

The objective of this study is to investigate the prognostic value of standard intermittent EEG based on the standardized ACNS terminology in a South Korean area where WSLT must not be performed. We also evaluate the performance of EEG findings combined with other clinical features during the early post-CA period for predicting favourable neurologic outcomes at 6 months after CA.

## 2. Materials and Methods

### 2.1. Study Design and Patients 

This retrospective, observational, registry-based cohort study was conducted at the emergency intensive care unit (ICU) of a tertiary care university-affiliated teaching hospital in Korea. Data were extracted from an OHCA registry, containing the prospectively collected data of consecutive adult patients ( ≥ 18 years) with OHCA since January 2010 [14,15]. The Institutional Review Board of the University of Ulsan College of Medicine reviewed and approved the study protocol (No. 2019–1883), and informed consent was waived because of the retrospective nature of the study. 

We included comatose patients with successfully resuscitated non-traumatic OHCA who were subjected to TTM between January 2010 and December 2018 and underwent routine EEG during the TTM period (within 72 h after the return of spontaneous circulation (ROSC)). We excluded patients who could not undergo EEG assessment within 72 h after ROSC or who showed poor-quality EEG data. The aim was that standard EEG examination was to be performed as soon as possible after ICU admission, but it was delayed whenever the patient was admitted to the ICU on weekends or after daytime (between 8 AM and 6 PM) on weekdays, owing to practical issues. All patients were followed up for 6 months after experiencing CA and were also subjected to neurologic assessment according to their Cerebral Performance Category (CPC) score. 

### 2.2. Management and Data Collection 

All patients were treated according to the then-current advanced cardiac life support guidelines [3,16]. TTM was performed for all unconscious patients using an Arctic Sun Energy Transfer Pad (Medivance Corp., Louisville, CO, USA), and the target temperature (33 °C or 36 °C) was maintained for 24 h. Considering the potential risks and benefits of temperature intervention, the patients who had intractable arrhythmia, clinically significant bleeding, or intractable hemodynamic instability were treated with the target temperature of 36°C; and the others with a target temperature of 33 °C. After 24 h, patients were rewarmed at a rate of 0.25°C/h following the maintenance of normothermia (37.0 °C) to prevent rebound fever for 72 h after ROSC. The temperature was monitored using an esophageal temperature probe. A combination of propofol, remifentanil, morphine, midazolam and fentanyl was used for sedation and analgesia. If necessary, a neuromuscular blocking agent was administered to control shivering during the induction period or to prevent respiratory dyssynchrony between the ventilator and the patient [17]. Patients with seizure activity observed on EEG were treated with valproate or levetiracetam. A 30-min scalp EEG was performed by the Stellate EEG system, in which 21 electrodes were placed according to the international 10–20 electrode system (Fp1-2, F7-8, T7-8, P7-8, F3-4, C3-4, P3-4, O1-2, Fz, Cz, Pz), with a sampling rate of 200 Hz and a 0.1-Hz high-pass filter. All patients received standard intensive care according to institutional protocols. WSLT was legally prohibited in South Korea during the study period and all the patients received treatment at the institution until death or recovery. 

**Figure 1:**
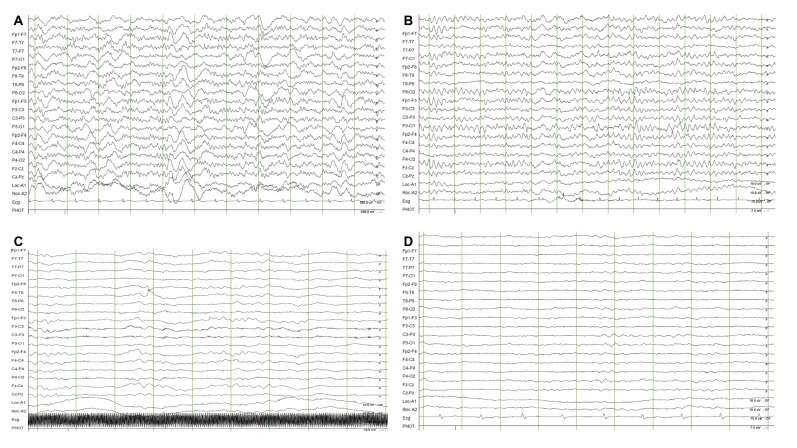
Example of predominant background electroencephalography frequency in out-of-hospital cardiac arrest survivors subjected to targeted temperature management. (**A**) Predominant alpha waves; (**B**) predominant theta waves; (**C**) predominant delta waves; (**D**) undetermined background electroencephalography.

Demographic and clinical data, including age, sex, previous medical history, resuscitation profiles and interventions were obtained. For this study, two board-certified epileptologists (M.K., Y.S.K) reviewed and interpreted the EEG recordings, blinded to the outcome. Background EEG were categorized according to the predominant frequency (alpha, theta, delta waves; Figure 1), voltage (attenuation or suppressed, <10 uV; low voltage, 10–20 uV; normal; >20 uV), other factors (reactivity, stage II sleep transients, burst suppression or burst attenuation) and superimposed findings, such as sporadic epileptiform, were also assessed according to ACNS guidelines [10]. An undetermined frequency was defined as discontinuous background (10–49% periods of suppression/attenuation or 50–99% periods of suppression or attenuation with burst suppression/attenuation) with attenuated/suppressed voltage or burst suppression/attenuation. All discordant EEG findings were discussed until a consensus was reached. The interrater agreement was 99% and only two cases were discordant. The primary endpoint was a favourable neurological outcome at 6 months, defined as a Cerebral Performance Category (CPC) score of one (no significant impairment) or two (moderate impairment but able to complete activities of daily living). The date of each patient’s death was obtained by reviewing the electronic medical records or using the National Health Insurance Service in South Korea. The neurologic outcome of survivors was determined at 1 and 6 months by reviewing the electronic medical records of hospitalized patients or by standardized follow-up telephone interviews with the patient or a family member as a primary caregiver by an investigator (YJK). 

### 2.3. Statistical Analysis

Because of their non-normal distribution, continuous variables are presented as median values with interquartile ranges (IQRs) using the Kolmogorov–Smirnov test. Categorical variables are expressed as an absolute number and percentage. Comparisons of demographic and clinical characteristics between the favourable and unfavourable neurologic outcome groups were performed using the Mann–Whitney U-test for continuous variables and the cthi-square test for categorical variables. On the basis of clinical judgment, clinical features and EEG findings of potential prognostic value were first examined at the baseline using univariate logistic analysis with a cut-off *p* value of <0.05. The optimal cut-off value of resuscitation duration and age was determined using the Youden index, which defines the cut-off in terms of the maximal sum of sensitivity and specificity. We used a backwards stepwise approach, sequentially eliminating variables with *p* value of >0.10 to build a final model. The results of the multivariate logistic regression analyses were summarized by estimating the odds ratios (ORs) and 95% confidence intervals (CI). The Hosmer–Lemeshow test for the logistic regression model was performed. Sensitivity, specificity, positive predictive value (PPV) and negative predictive value (NPV) for favourable neurological outcomes at 1 month were calculated. Two-tailed *p* values of <0.05 were considered to be statistically significant. All statistical analyses were performed using IBM SPSS Statistics for Windows, version 21.0 (IBM Corp., Armonk, NY, USA).

## 3. Results

**Figure 2:**
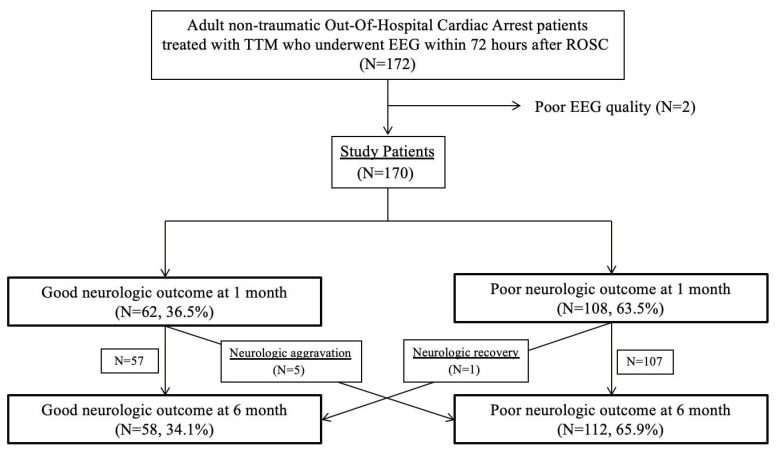
Patient flow diagram. Abbreviations: targeted temperature management (TTM); electroencephalography (EEG); return of spontaneous circulation (ROSC).

Among 277 non-traumatic comatose OHCA survivors treated with TTM, 170 patients who underwent standard intermittent EEG during TTM were finally included (Figure 2). Between 1 and 6 months, one patient showed neurologic recovery, while five patients showed unfavourable neurologic outcomes or death at 6 months after CA. Finally, 170 patients were categorized into the favourable (*n* = 58, 34.1%) and poor (*n* = 112, 65.9%) neurological outcome groups, respectively.

The demographic and clinical characteristics of the patients are summarized in Table 1. Patients in favourable neurologic outcome groups were younger (median, 54.5 vs. 62.0 years; *p* < 0.001) and had a higher rate of initial shockable rhythm (67.2% vs. 24.1%; *p* < 0.001) as well as a higher likelihood of having a witnessed CA (84.5% vs. 69.6%; *p* = 0.035). Resuscitation duration was shorter in the favourable neurologic outcome group (median, 11.5 vs. 26.5 min; *p* < 0.001). EEG timing did not differ between two groups: the median time to EEG examination was 22 h. Propofol was used more frequently for the patients in the favourable neurologic outcome group (96.6% vs. 74.1%; *p* < 0.001), without a significant dose difference.

The EEG findings are presented in Table 2. Alpha and theta waves were more prevalent in the favourable neurologic outcome group, whereas undetermined background activity was predominant in the poor neurologic outcome group (*p* < 0.001). The majority of patients in the favourable neurologic outcome group had a normal voltage (83.9%). Burst suppression/burst attenuation (12.9%), reactivity to pain stimuli (16.5%) and rhythmic delta activity (24.7%) were not frequently reported findings, but there were significant differences between the favourable and unfavourable neurologic groups.

In univariate analysis, age, initial shockable CA rhythm, presence of a witness during CA, resuscitation duration and EEG findings, such as predominant background EEG frequency and voltage, burst suppression or burst attenuation, reactivity to pain stimuli and rhythmic delta activity, were all associated with favourable neurologic outcomes (Table 3). After conducting multivariate logistic regression analysis, we identified that age, initial shockable CA rhythm, resuscitation duration and background EEG frequency were significant prognostic factors. Finally, an age of <65 years (adjusted OR, 7.973; 95% CI, 2.678–23.741; *p* < 0.001), initial shockable CA rhythm (adjusted OR, 7.965; 95% CI, 2.865–22.145; *p* < 0.001), <20-min resuscitation duration (adjusted OR, 5.297; 95% CI, 1.868–15.020; *p* = 0.002) and predominant background EEG frequency with dominant alpha and theta waves (adjusted OR, 13.030; 95% CI, 4.687–36.224; *p* < 0.001) were associated with favourable neurologic outcomes at 6 months.

Diagnostic performances of the combination of predominant background EEG frequencies and other clinical features for predicting favourable neurologic outcomes are summarized in Table 4. Background EEG frequency with dominant alpha and theta waves predicted favourable neurologic outcomes with a sensitivity of 86.21%, a specificity of 75.00%, a positive predictive value of 64.10% and a negative predictive value of 91.30%. The presence of all four criteria predicted favourable neurologic outcomes with a specificity of 100.0% and a positive predictive value of 100.0%.

## 4. Discussion

This registry-based cohort study adds to the current understanding regarding the use of standard intermittent EEG as an early prognostic tool for comatose OHCA survivors who underwent EEG during TTM in areas where WSLT was legally prohibited. Standard EEG examination revealed that 45.9% (78/170) of the non-traumatic OHCA survivors had dominant alpha and theta waves, and the presence of these EEG findings indicated a favourable neurologic outcome with a sensitivity of 86.21% and a negative predictive value of 91.30% under the effect of sedative medication during TTM. Furthermore, background EEG frequency combined with other clinical features, including age, initial shockable rhythm and resuscitation duration, led to an increase in predictive performance for favourable neurologic outcomes, with a sensitivity of 95.2% and a specificity of 100%. 

### 4.1. The Prognostic Value of EEG Findings: Compared with Previous Studies 

Our study demonstrated that the presence of dominant alpha and theta waves, even documented in intermittent EEG examinations, would provide reliable information regarding the probability of achieving favourable neurological outcomes. Although several studies have suggested the prognostic value of background continuity using continuous or intermittent EEG monitoring, they mainly focused on the prognostic value of EEG findings rather than adjusting other clinical prognostic features in OHCA patients [13,18,19,20,21]. Previous studies categorized EEG findings into highly malignant, malignant and benign patterns in CA patients with TTM and evaluated the prognostic value in predicting unfavourable neurological outcomes. In the sub-study of a TTM trial, containing 103 patients with intermittent EEG, it was found that highly malignant EEG, after rewarming, reliably predicted poor outcomes in half of the patients, without false predictions [18]. Furthermore, Rossetti et al. showed that highly malignant EEG was associated with mortality, with a false positive rate of 1.5% [11]. Standardized EEG categorization after CA shows a strong correlation with other outcome predictors [22]. In our study, the background EEG frequency was the only significant factor among the prespecified “benign” EEG patterns based on the ACNS nomenclature after adjusting other clinical features [10]. Other previous studies that analyzed continuous EEG records also demonstrated that EEG background patterns could help physicians to predict both favourable and unfavourable neurologic outcomes in the very early phase (at 24 h after ROSC) [20,21]. Ruijter et al. analyzed the continuous EEG data of comatose CA patients for 72 h using quantitative EEG techniques, and they found the combination of the background continuity index and the burst-suppression amplitude ratio could predict unfavourable neurologic outcomes with 100% specificity at 12 h after CA [13]. Despite the prognostic power of continuous EEG monitoring in OHCA patients, the interpretation and application of 24-hour continuous EEG monitoring is not commonly available and our strategy for evaluating the predominant background EEG frequency, categorized into generalized delta activity of 1–3 cycles per second (Hz), theta activity of 4–7 Hz and alpha activity of 8–12 Hz, was more practical, simple and relatively objective. 

### 4.2. The Prognostic Value of Background EEG Frequency

The background EEG frequency with dominant alpha and theta waves was a powerful predictor (adjusted OR for favourable neurologic outcome of 13.030) with high sensitivity (86.21%) and negative predictive value (91.30%). In our study, 36% (28/78) of the patients with dominant alpha and theta waves had unfavourable neurologic outcomes. Our results can be interpreted in two aspects. Firstly, despite the background continuity of the alpha or theta activity on EEG, specific coma patterns known as the alpha and theta comas with monotonous, invariable frontal predominance rhythms were also differentiated from the alpha and theta activities [23]. These EEG findings indicate an unfavourable prognosis [23]. Secondly, the restoration timing is another important factor. The median time to EEG examination in our study was 22 h, but some studies suggested that regaining background continuity within 24 h was a key prognostic finding for a favourable neurologic outcome [13,24]. For those 28 patients, 16 (57%) patients underwent EEG examination after 24 h following ROSC. These results are supported by a recent study from Duez et al. [25]. They found no differences between prognostication at 24 h compared to 48 h, measured via the specificity and sensitivity of EEG categories, but when using EEG reactivity, prognostication was the best at 24 h compared to 48 h [25].

### 4.3. Other Clinical Prognostic Factors Combined with EEG Findings

We also identified three clinical factors, namely an age of <65 years, initial shockable rhythm and resuscitation duration of <20 min, which were associated with favourable neurologic outcomes at 6 months. Shockable arrest rhythm, young age and short resuscitation duration are well-known favourable prognostic factors in CA survivors. Patients with dominant alpha and theta waves on EEG or shockable initial rhythms are predicted to have a favourable neurologic outcome with a sensitivity of 94.8%, whereas those with EEG findings as well as the three above-mentioned clinical factors are predicted to have a favourable neurologic outcome with a specificity of 100%. Our findings suggested that a combination of dominant alpha and theta waves on early standard EEG during TTM, and the other three clinical features, could have practical implications in the identification of comatose OHCA survivors with favourable neurologic outcomes. Additionally, one patient who had unfavourable neurologic outcomes at 1 month (CPC 3) showed neurologic recovery at 6 months (CPC 1). This 60-year male patient had predominant theta waves on his EEG without an initial shockable rhythm. Many previous studies demonstrated that young CA survivors had a higher likelihood for neurological recovery, despite their presumed unfavourable prognosis, and therefore WLST should be carefully considered for CA survivors of a young age [23,26,27].

### 4.4. Limitations 

The results of our study should be interpreted in the context of the following limitations. First, the timing of standard EEG examination could affect the outcome. Although, at our center, standard EEG is usually performed as soon as possible after ICU admission, the median EEG examination period was 22 h because of its unavailability on a 24/7 basis. Nevertheless, our heterogeneous EEG timing during TTM reflected the real-world situation and can be generalizable to other settings. Second, the likelihood of clinically important EEG findings and transitions or the different effects of sedative agents should be considered. Moreover, the heterogeneity of sedative sedation regimes should be considered. However, recent studies have revealed that sedative agents have a minimal effect on the predictive power of EEG [13,28]. Third, the long study period between 2010 and 2018 accompanied by changes in treatment guidelines might affect the outcome of OHCA survivors. Fourth, this study was based on data from a single institution and had an observational study design, which limited generalization and gave rise to an unmeasurable confounding bias. Finally, despite our sample size being relative larger than that of previous studies, there was an inevitably high potential of random effect generation resulting from such a small sample.

## 5. Conclusions

This study demonstrated that standard intermittent EEG findings of predominant background frequency during the early post-CA period in comatose OHCA patients could be an early prognostic tool to identify patients with favourable neurologic outcomes. Furthermore, the EEG findings and the findings of three other clinical features (age < 65 years, initial shockable rhythm and resuscitation duration of <20 min) have practical implications for the early prognostication of favourable neurologic outcomes. Further prospective multi-center studies are warranted to validate the predictive value of the early standard intermittent EEG findings proposed in this study.

## Figures and Tables

**Table 1:** Demographic and clinical characteristics of patients with out-of-hospital cardiac arrest treated with targeted temperature management according to neurologic outcome at 6 months.

Characteristics	Total (*n* = 170)	Favourable Neurologic Outcome (*n* = 58)	Unfavourable Neurologic Outcome (*n* = 112)	*p*-Value
Age, years	60.0 (45.8–71.0)	54.5 (39.0–64.3)	62.0 (49.0–73.0)	<0.001
Age < 65 years	107 (62.9%)	48 (82.8%)	59 (52.7%)	<0.001
Male	113 (66.5%)	42 (72.4%)	71 (63.4%)	0.238
Previous medical history				
Hypertension	58 (34.1%)	14 (24.1%)	44 (39.3%)	0.048
Diabetes mellitus	43 (25.3%)	8 (13.8%)	34 (31.5%)	0.013
Acute myocardial infarction	8 (4.7%)	1 (1.7%)	7 (6.3%)	0.267
Congestive heart failure	12 (7.1%)	4 (6.9%)	8 (7.1%)	>0.999
Chronic kidney disease	20 (11.8%)	3 (5.2%)	17 (15.2%)	0.055
Malignancy	13 (7.6%)	3 (5.2%)	10 (8.9%)	0.382
Arrest characteristics				
Witnessed	127 (74.7%)	49 (84.5%)	78 (69.6%)	0.035
Bystander CPR	120 (70.6%)	39 (67.2%)	81 (72.3%)	0.491
Initial shockable rhythm	66 (38.8%)	39 (67.2%)	27 (24.1%)	<0.001
No flow time, min	0.0 (0.0–3.0)	0.0 (0.0–4.0)	0.0 (0.0–3.0)	0.778
Resuscitation duration, min	22.0 (10.0–35.3)	11.5 (6.0–20.8)	26.5 (16.0–39.5)	<0.001
Time from ROSC to target temperature, hours	5.52 (3.93–7.43)	5.75 (4.88–7.97)	5.00 (3.76–7.47)	0.069
Target temperature				0.679
33 °C	150 (88.2%)	52 (89.7%)	98 (87.5%)	
36 °C	20 (11.8%)	6 (10.3%)	14 (12.5%)	
Phases of TTM at EEG examination				0.730
Induction	15 (8.8%)	6 (10.3%)	9 (8.0%)	
Maintenance	92 (54.1%)	32 (55.2%)	60 (53.6%)	
Rewarming	20 (11.8%)	8 (13.8%)	12 (10.7%)	
Normothermia	43 (25.3%)	12 (20.7%)	31 (27.7%)	
Time from ROSC to EEG examination, hours	22.0 (12.8–40.3)	21.0 (11.0–36.8)	22.0 (13.3–42.5)	0.581
Treated sedatives				
Propofol	139 (81.8%)	56 (96.6%)	83 (74.1%)	<0.001
Max. propofol rate, mg/kg/h	3.0 (2.0–4.0)	3.0 (2.0–4.0)	3.0 (1.5–4.0)	0.166
Remifentanil	87 (51.2%)	34 (58.6%)	53 (47.3%)	0.162
Max remifentanil rate, µg/kg/h	0.2 (0.1–0.2)	0.2 (0.1–0.2)	0.2 (0.1–0.2)	0.258
Morphine	54 (31.8%)	21 (36.2%)	33 (29.5%)	0.371
Max morphine rate, mg/h	2.5 (1.9–3.0)	3.0 (2.0–3.5)	2.0 (1.0–3.0)	0.348
Midazolam	37 (21.8%)	14 (24.1%)	23 (20.5%)	0.589
Max midazolam rate, mg/kg/h	0.10 (0.08–0.16)	0.08 (0.04–0.11)	0.10 (0.08–0.20)	0.219
Fentanyl	2 (1.2%)	0 (0%)	2 (1.8%)	0.548
Treated neuromuscular blocking agent	35 (20.6%)	15 (25.9%)	20 (17.9%)	0.221

Values are expressed as median (interquartile ranges) or *n* (%) as appropriate. Abbreviations: CPR, cardiopulmonary resuscitation; ROSC, return of spontaneous circulation; EEG, electroencephalography.

**Table 2:** Standard intermittent electroencephalography findings based on the standardized terminology of the American Clinical Neurophysiology Society.

Characteristics	Total (*n* = 170)	Favourable Neurologic Outcome (*n* = 58)	Unfavourable Neurologic Outcome (*n* = 112)	*p*-Value
EEG background frequency				<0.001
Dominant alpha waves	61 (35.9%)	36 (62.1%)	25 (22.3%)	
Dominant theta waves	17 (10.0%)	14 (24.1%)	3 (2.7%)	
Dominant delta waves	15 (8.8%)	2 (3.4%)	13 (11.6%)	
Undetermined	77 (45.3%)	6 (10.3%)	71 (63.4%)	
EEG background voltage				<0.001
Attenuation or Suppressed (<10 μV)	64 (37.6%)	4 (6.9%)	60 (53.6%)	
Low voltage (10–20 μV)	13 (7.6%)	4 (6.9%)	9 (8.0%)	
Normal (>20 μV)	93 (54.7%)	50 (86.2%)	43 (38.4%)	
Other background EEG findings				
Burst suppression or Burst attenuation	22 (12.9%)	2 (3.4%)	20 (17.9%)	0.008
Reactivity to pain stimuli	28 (16.5%)	16 (27.6%)	12 (10.7%)	0.005
Stage II Sleep transients	9 (5.3%)	6 (10.3%)	3 (2.7%)	0.064
Epileptic form discharge				
Spike and wave or Sharp and wave	14 (8.2%)	4 (6.9%)	10 (8.9%)	0.774
Rhythmic delta activity	42 (24.7%)	27 (46.6%)	15 (13.4%)	<0.001

Abbreviations: EEG, electroencephalography.

**Table 3:** Univariate and multivariate logistic regression analysis for a 6-month favourable neurologic outcome.

Characteristics	Crude OR	95% CI	*p*-Value	Adjusted OR	95% CI	*p*-Value
Age, years				0.950	0.921–0.980	0.001
Female	0.660	0.330–1.318	0.239			
Initial shockable rhythm	6.462	3.213–12.996	<0.001	7.158	2.779–20.334	<0.001
Witnessed	2.373	1.048–5.372	0.038			
Bystander CPR	0.786	0.395–1.562	0.491			
No flow time, min	1.011	0.953–1.072	0.725			
Resuscitation duration, min	0.959	0.938–0.982	<0.001	0.966	0.939–0.993	0.015
Predominant background EEG frequency						
Undetermined	Reference			Reference		
Dominant alpha waves	17.040	6.414–45.271	<0.001	9.576	3.087–29.703	<0.001
Dominant theta waves	55.222	12.325–247.425	<0.001	31.843	5.861–173.017	<0.001
Dominant delta waves	1.821	0.331–10.026	0.491	2.333	0.347–15.691	0.384
Voltage of background EEG frequency						
Normal	Reference					
Attenuation or Suppressed (<10 μV)	0.057	0.019–0.171	<0.001			
Low voltage (10–20 μV)	0.382	0.110–1.329	0.130			
Other EEG findings						
Burst suppression or Burst attenuation	0.164	0.037–0.730	0.018			
Reactivity to pain stimuli	3.175	1.383–7.286	0.006			
Stage II Sleep transients	4.192	1.009–17.426	0.049			
Epileptic form discharge						
Spike and wave or Sharp and wave	0.756	0.226–2.522	0.649			
Rhythmic delta activity	5.632	2.662–11.919	<0.001			

Abbreviations: odds ratio (OR); confidence interval (CI); cardiopulmonary resuscitation (CPR); electroencephalography (EEG).

**Table 4:** Predictive value of clinical and EEG findings for favourable neurologic outcome (favourable neurologic outcome event/total patients = 58/170).

Variables	Favourable Neurologic Outcome/Patient Numbers	Sensitivity	Specificity	PPV	NPV	Accuracy
EEG pattern	50/78	86.2%	75.0%	64.1%	91.3%	78.8%
EEG pattern and/or rhythm	55/100	94.8%	59.8%	55.0%	95.7%	71.8%
EEG pattern + age	40/53	69.0%	88.4%	75.5%	84.6%	81.8%
EEG pattern + rhythm	34/44	58.6%	91.1%	77.3%	81.0%	80.0%
EEG pattern + age + rhythm	27/31	46.6%	94.4%	87.1%	77.7%	79.4%
EEG pattern + age + rhythm + resuscitation duration	17/17	29.3%	100.0%	100.0%	73.2%	75.9%

EEG pattern indicates a predominant background EEG frequency with dominant alpha and theta waves. Rhythm indicates initial shockable cardiac arrest rhythm. Age indicates an age under 65 years old. Resuscitation duration indicates <20 minutes. Abbreviations: positive predictive value (PPV); negative predictive value (NPV).
